# Comparative effectiveness of two different interbody fusion methods for transforaminal lumbar interbody fusion: cage versus morselized impacted bone grafts

**DOI:** 10.1186/s12891-015-0675-2

**Published:** 2015-08-19

**Authors:** Chaoliang Lv, Xianzhou Li, Haicheng Zhang, Junrong Lv, Hongmei Zhang

**Affiliations:** Jining No. 1 People’s Hospital, No. 6 Jiankang road, Jining, Shandong Province China

**Keywords:** Interbody fusion, Bone graft, Cage, Lumbar spinal stenosis

## Abstract

**Background:**

The use of an interbody fusion device (cage) to assist fusion and increase intervertebral stability is widely supported. We applied the morselized impacted bone graft method without using a cage in a single level interbody fusion with encouraging medium-term clinical results. The purpose of this paper is to compare the clinical and radiological results of local bone grafts with a cage to morselized impacted bone grafts without cage, in patients undergoing transforaminal lumbar interbody fusion (TLIF) surgery.

**Methods:**

One hundred eighty-nine consecutive patients who underwent TLIF in our hospital were evaluated from July 2009 to July 2012. Eighty-four patients received TLIF and local bone graft with one polyetheretherketone (PEEK) cage, while 96 patients received the TLIF with local morselized impacted bone grafts without a cage. The clinical data and perioperative parameters of the patients in the two groups were recorded and compared.

**Results:**

The mean follow-up time was 35 months. There were no significant differences in operation time and blood loss between the two groups. Single-level fusion was performed in all patients. There were no statistically significant differences between the two groups, according to the preoperative or postoperative Oswestry Disability Index (ODI) score. No statistically significant differences in fusion rate were observed between the two groups. At the final follow-up, the ratio of the disc height to vertebral height (HR) was not significantly different between the two groups.

**Conclusion:**

Morselized impacted bone graft is as beneficial as local bone grafts with a cage for TLIF. Since the no cage procedure is less expensive, the morselized impacted bone graft is an affordable choice for single level TLIF, especially in less developed regions.

## Background

The purpose of the lumbar spine fusion is to obtain a solid arthrodesis in order to alleviate pain [1]. Transforaminal Lumbar Interbody Fusion (TLIF) is gaining wide acceptance for the treatment of the degenerative lumbar spine and has proven advantages in the restoration of the disc height, disc stabilization, nerve root decompression, and reinforcement of the anterior spinal column [2–4].

In TLIF, success of the fusion relies mainly on the type of instrumentation and the bone graft material. The use of an interbody fusion device (cage) to assist fusion and increase the stability of the construct is widely supported [5, 6]. The bone grafts used in TLIF should have an inherent osteogenic capacity and good mechanical strength [7]. Autogenous iliac bone is the most ideal graft in terms of osteogenic capacity, but is associated with donor site pain and additional surgical invasion [8]. A local morselized bone graft composed of the lamina as well as the articular and spinous processes that were obtained during posterior decompression, has proven to be an ideal alternative material [9]. At present, a cage filled with local morselized bone graft is widely used in TILF procedures. Segmental pedicle screw fixation is additionally performed in order to prevent iatrogenic instability of the posterior joint.

Morselized impacted bone grafting is often performed in limb lesions in order to provide sufficient biomechanical strength. Patients’ medical expenses were greatly reduced by savings accrued by not having to spend for the cost of the cage

The authors conducted a retrospective comparative study of morselized impacted bone graft TLIF and cage TLIF in order to evaluate the clinical and radiological outcomes, as well as to determine any objective benefits of morselized impacted bone graft TLIF.

## Methods

### Patient selection

Among the 189 enrolled cases, 9 patients were lost to follow up. We retrospectively studied 180 patients treated with TLIF between July 2009 and July 2012 at an average follow up of 35 months. While 84 patients received TLIF with local bone graft combined with one PEEK cage (group 1), 96 patients received the TLIF with local morselized impacted bone grafts without a cage (group 2). Patients with lumbar spinal stenosis associated with low back and leg pain at L4-5 or L5-S1 were included in this study. MRI (magnetic resonance imaging) was used for diagnosis and only single level fusion was performed. The symptomatic indications included disabling back pain that resulted in lifestyle alterations and failure of nonoperative treatment for more than 6 months. Patients with spondylolisthesis, spinal infection, severe osteoporosis, and previous spinal fusion operations were excluded from the study.

Written consent was obtained from the patient for publication of study. The research were approved by the Ethical Committee of Jining No. 1 People’s Hospital, Jining, Shandong Province, China.

### Surgical technique

The spine was approached through a classic posterior midline incision and subperiosteal muscular detachment. Resection of the spinous process and laminae was performed using an osteotome. The facetectomy side was chosen according to the subject’s leg pain symptoms. The harvested local bone was cut into small pieces for later use. An annulotomy was created and subtotal discectomy was performed. The hyaline cartilage of the endplates was removed until the surgeon was satisfied with endplate preparation. For fusion in group 1, a PEEK cage packed with autologous local bone was used and additional autograft locally harvested from decompression was packed behind the cage. In group 2, smaller pieces were inserted and impacted into the intervertebral disc space using a bone-grafting funnel. The key point of the procedure in group 2 is the continued impaction with bone graft in order to achieve closer contact between the bone and bone graft bed and sufficient biomechanical strength.

Lastly, the pedicle screws were inserted with rods in all cases. Standard wound closure was performed following hemostasis. In all cases, a lumbosacral orthosis was used from the 3rd postoperative day until the 4th-5th postoperative week when the patient was walking independently. The operation time, amount of blood loss, and the perioperative complications were recorded.

### Assessments

**Fig. 1:**
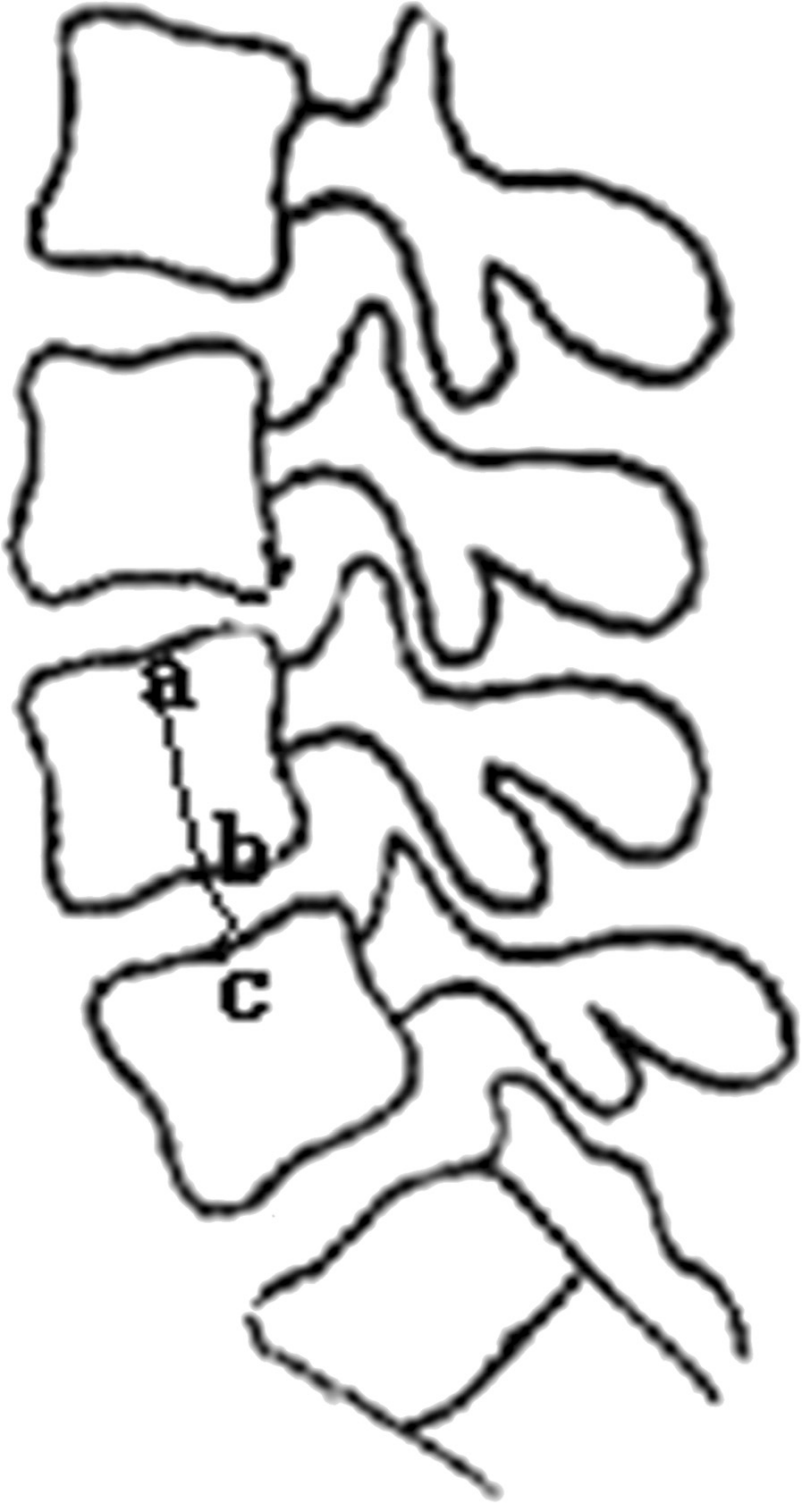
An illustration of the method for measuring the ratio of disc to vertebra heights (*HR*) with, “a”, “b” and “c” as endplate midpoints. HR = bc/ab

The clinical function evaluation was based on the preoperative ODI and at the last follow up day. Lateral plain radiographs taken at the preoperative, immediate postoperative and at the last follow-up period were compared for radiological assessment. The disc height (DH) was calculated as the ratio between the disc height and the height of the superior vertebral body. The height of the vertebral body and the disc were measured by the link between the superior and inferior endplate midpoints. The ratio of disc height to vertebral height was defined as HR (Fig. 1).

Fusion was determined by computed tomography (CT) scanning and ranked according to 5 grades based on the anterior fusion criteria described by Brantigan et al [10] (grades 1 and 2, not fused; grade 3, uncertain; grades 4 and 5, fused) (Table 1). Grades 4 and 5 are considered as fused. All patients underwent 0.75 mm thin-section helical CT scanning with sagittal and coronal views of the involved lumbar segments. Two radiologists who were blinded to the patients’ clinical data performed all evaluations independently.

**Table 1:** Brantigan fusion grading criteria

Grade 1	Unfused	Obvious radiographic Pseudarthrosis based on collapse of the construct, loss of disk height,vertebral slip,broken screws,cage displacement.
Grade 2	Probable unfused	Probable radiographic pseudarthrosis based on significant resorption of the bone graft, or a major lucency or gap visible in the fusion area.
Grade 3	Uncertain	Bone graft is visible in the fusion area at approximately the density originally achieved surgically. A small lucency or gap may be visible involving a portion of the fusion area with at least half of the graft area showing no lucency between the graft bone and vertebral bone.
Grade 4	Probable fused	Bone bridges the entire fusion area with at least the density originally achieved intraoperatively. No lucency between the donor bone and vertebral bone should be present.
Grade 5	Fused	The bone in the fusion area is radiographically denser and more mature than originally achieved intraoperatively. No lucency could be detected between the graft bone and cage with vertebral bone.

### Statistical analysis

Statistical analysis was performed using the SPSS 13.0 (SPSS Inc., Chicago, Illinois) software. Data were expressed as mean ± standard deviation (SD). The t- and Chi-square tests were used to compare results of groups 1 and 2. A *P*-value of less than 0.05 was considered statistically significant.

## Results

### Patient population

Among the 189 enrolled cases, 9 were lost to follow-up. There were no significant differences in clinical baseline data, including age, sex, ODI, and levels between the two groups (Table 2). The mean follow-up duration was 35 months (range 22–48 months).

**Table 2:** Baseline clinical characteristics of the patients in groups 1 and 2

	Group 1	Group 2	Statistics
No. of cases	84	96	
sex: M/F	32/52	37/59	NS^a^
Mean age(years)	51(31–68)	53(29–69)	NS^a^
Meanfollow-up(years)	43(36–49)	30(24–40)	NS^a^
ODI	42(32–65)	45(33–67)	NS^a^
levels (*n*)			
L4/5	61	67	NS^a^
L5/S1	23	29	NS^a^

^a^Not significant; chi-square test for sex ratio, t-test for age follow-up, ODI, and levels

### Perioperative parameters

The mean operating time was 125 ± 18 and 118 ± 15 min in groups 1 and 2, respectively. The mean blood loss was 330 ± 45 mL (ranged 300–800 mL) and 310 ± 35 mL in groups 1 and 2. There were no significant differences in operation time or blood loss between the two groups (*P* = 0.71 and *P* = 0.69).

### Clinical and radiological outcome

Single-level fusions were done in all patients. There were no statistical differences in the pre- or postoperative ODI, as well as changes between the pre- and postoperative ODI scores in the 2 patient groups (Table 3).

**Table 3:** Comparison of pre- and postoperative changes (mean and statistical significance) between the 2 patient groups

		Group 1			Group 2		Statistics
	Preop	Postop	Δ	Preop	Postop	Δ	
ODI	42(32–65)	21(2–48)	24(–23–62)	45(33–67)	19(0–51)	26(–16–59)	NS^a^

^a^Not significant (t-test). Δ: change from pre- to postoperative value. ODI: Oswestry Disability Index. Postop: at the last follow up

**Fig. 2:**
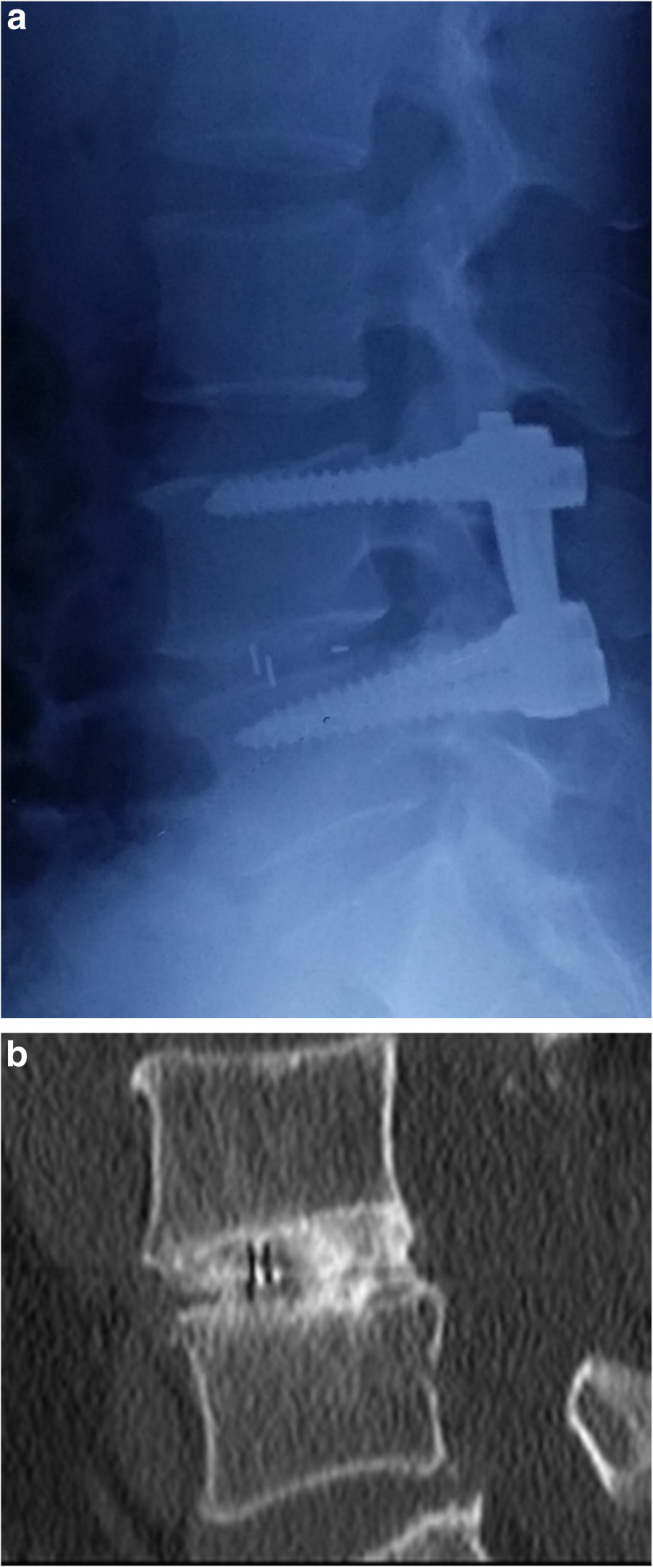
**a** Lateral view of a patient who underwent TLIF with a PEEK cage at the first postoperative week. **b** CT scan images at the twenty-fourth postoperative month demonstrating a stable Grade 5 bony fusion at the L4/5 level, as described by the anterior fusion criteria defined by Brantigan

**Fig. 3:**
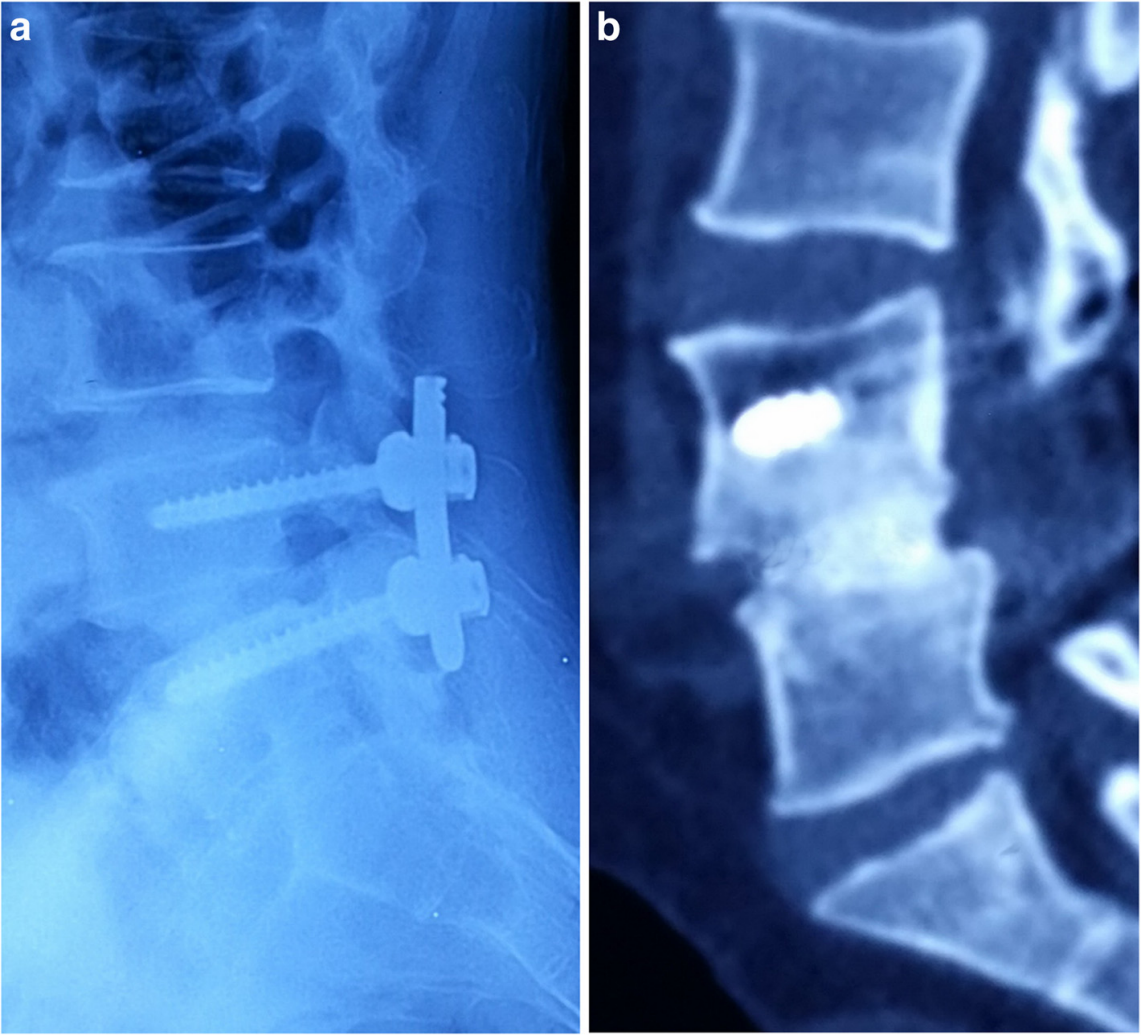
**a** Lateral view of a patient who underwent TLIF with morselized impacted bone graft without a cage at the first postoperative week. **b** CT scans images at the twenty-sixth postoperative month demonstrating stable Grade 5 bony fusion at the L4/5, as described by the anterior fusion criteria defined by Brantigan

Fusion status in all patients at the follow-up time was assessed by CT scans, according to the anterior fusion criteria described by Brantigan. Grades 4 and 5 are considered fused and were observed in 79 out of 84 patients (94.05 %) and 91 of 96 patients (94.79 %) in groups 1 and 2, respectively. There were no significant differences between the two groups (*P* = 0.792) (Figs. 2 and 3).

The mean disc height to vertebral body ratio (HR) was restored and preserved in both of the groups. At the final follow-up, HR was not significantly different between the two groups (Table 4).

**Table 4:** Comparison of HR measurements between the two patient groups (mean ± SD)

	Group 1		Group 2	
	L4/5	L5/S1	L4/5	L5/S1
HR				
Preop	0.37 ± 0.09	0.34 ± 0.18	0.31 ± 0.19	0.34 ± 0.12
Postop	0.46 ± 0.11	0.45 ± 0.14	0.45 ± 0.07	0.45 ± 0.13
Final follow-up	0.45 ± 0.09	0.44 ± 0.16	0.44 ± 0.13	0.44 ± 0.09
*P* value 1	0.000	0.000	0.000	0.000
*P* value 2	0.521	0.325	0.238	0.104

*P* value 1: the difference between the pre- and postoperative HR

*P* value 2: the difference between the postoperative and the final follow-up HR

No significant differences between groups 1 and 2 at every period

HR: ratio of the height of the disc to the height of the superior vertebral body

## Discussion

In TLIF surgery, bone graft material plays a key role in getting solid interbody fusion. The iliac crest has previously been considered as an ideal source of graft, in terms of quantity and quality. An iliac crest bone graft facilitates rapid bone union, but increases the risk of donor site pain, infection, excessive blood loss, pelvic fracture, an additional skin incision, and lengthy operative time. In contrast, a local bone graft consisting of the lamina as well as articular and spinous processes obtained from decompression, can shorten the operative time and reduce blood loss without any problems at the donor site. Many studies have reported that local bone chips obtained from decompression and used as bone grafts demonstrate comparable fusion rates with the iliac crest [11, 12]. Therefore, the local bone has already become a frequently used bone graft substitute for the iliac crest.

As an interbody fusion device, the cage was often used for interbody fusion in order to restore the disc height in cases with collapsed degenerated discs as well as to provide immediate anterior load sharing support [13]. In the TLIF procedure, local bone obtained from decompression was morselized and then transplanted at the anterior portion of the intervertebral disc space. A cage filled with local morselized bone was subsequently inserted. We obtained good bone union with these procedures [14–16].

Previous studies have shown that cages reduced the contact area for bony fusion, which resulted in low fusion rates [17]. In addition, the cost of cages is expensive for patients from developing countries and regions. Furthermore, the complications related to using the cage, including retropulsion and migration as well as collapsed end plates, were reported occasionally [18]. Morselized impacted bone graft is often performed in limb lesions, which can supply sufficient biomechanical strength. In TLIF, we applied the morselized impacted bone graft method in interbody fusion without using a cage. Mechanical strength was greatly improved by impacting. Patients’ medical expenses were greatly reduced by not having to spend for the cost of the cage [19]. The morselized impacted bone grafts also enlarged the bone contact area, which in turn, reduced the risk for bony fusion. Medium-term clinical results were encouraging. However, few studies have compared local bone grafts with one cage to morselized impacted bone grafts without a cage, according to the clinical and radiological effects.

The purpose of this study was to compare perioperative parameters and medium-term clinical and radiological outcomes between patients treated with TLIF using one PEEK cage and those treated with TLIF without a cage with the same intervertebral device, and if possible, find any objective benefits of TLIF without cage.

Based on the results of our study, utilizing the morselized impacted bone graft is as beneficial as TLIF with the PEEK cage. In order to minimize the chance for bias, patients treated with only single level fusions were included. Similar clinical and radiological results were obtained from both of the groups for single level TLIF. However, some previous studies have reported greater improvement and better maintenance of disc space, vertebral height and the absence of collapse with the cage compared to the no cage group in ODI. Doctor Abdul reported that the increment in disc height and VAS score was significantly better in the cage group compared to the bone graft group. However, in his study, only local bone graft was used, which is less rigid and leads to collapse, pain, and disability [20]. The morselized impacted bone graft utilized in the present study is different from the local bone graft reported by Abdul. Continued impaction resulted in close contact between bone and bone graft bed and sufficient biomechanical strength [21]. Therefore, the mean disc height to vertebral body ratio was restored and preserved in the morselized impacted bone graft group, which was not significantly different from the cage group at the final follow up. In addition, the reliable biomechanical strength contributes to the good fusion rate and ODI scores.

## Conclusion

There were no significant differences in clinical and radiological results between the local bone graft with a cage and the morselized impacted bone graft groups, for one level TLIF. Additionally, the no cage procedure costs less than the cage procedure. Therefore, morselized impacted bone graft without a cage is another good option for cases of single level TLIF, especially in less developed regions.

## Abbreviations

TLIF: Transforaminal lumbar interbody fusion
PEEK: Polyetheretherketone
ODI: Oswestry Disability Index
MRI: Magnetic resonance imaging
CT: Computed Tomography
DH: The disc height
HR: The ratio of disc height to vertebral height
